# Anesthetic Propofol Overdose Causes Vascular Hyperpermeability by Reducing Endothelial Glycocalyx and ATP Production

**DOI:** 10.3390/ijms160612092

**Published:** 2015-05-27

**Authors:** Ming-Chung Lin, Chiou-Feng Lin, Chien-Feng Li, Ding-Ping Sun, Li-Yun Wang, Chung-Hsi Hsing

**Affiliations:** 1 Department of Anesthesiology, Chi Mei Medical Center, Liouying, 201, Taikang, Taikang Village, Liuying District, Tainan 736, Taiwan; E-Mail: mygegon@gmail.com; 2 Department of Medical Laboratory Science and Biotechnology, Chung Hwa University of Medical Technology, 89, Wenhwa 1st Street, Rende District, Tainan 717, Taiwan; 3 Department of Microbiology and Immunology, Graduate Institute of Medical Sciences, College of Medicine, Taipei Medical University, 250 Wuxing Street, Taipei 110, Taiwan; E-Mail: cflin2014@tmu.edu.tw; 4 Department of Pathology, Chi Mei Medical Center, 901 Zhonghua Road, Yongkang District, Tainan 710, Taiwan; E-Mail: angelo.p@yahoo.com.tw; 5 Department of Surgery, Chi Mei Medical Center, 901 Zhonghua Road, Yongkang District, Tainan 710, Taiwan; E-Mail: sdp0127@yahoo.com.tw; 6 Department of Anesthesiology, Chi Mei Medical Center, 901 Zhonghua Road, Yongkang District, Tainan 710, Taiwan; E-Mail: sindysea@hotmail.com; 7 Department of Anesthesiology, College of Medicine, Taipei Medical University, 250 Wuxing Street, Taipei 110, Taiwan

**Keywords:** propofol, mice, endothelial cells, glycocalyx, vascular permeability, ATP

## Abstract

Prolonged treatment with a large dose of propofol may cause diffuse cellular cytotoxicity; however, the detailed underlying mechanism remains unclear, particularly in vascular endothelial cells. Previous studies showed that a propofol overdose induces endothelial injury and vascular barrier dysfunction. Regarding the important role of endothelial glycocalyx on the maintenance of vascular barrier integrity, we therefore hypothesized that a propofol overdose-induced endothelial barrier dysfunction is caused by impaired endothelial glycocalyx. *In vivo*, we intraperitoneally injected ICR mice with overdosed propofol, and the results showed that a propofol overdose significantly induced systemic vascular hyperpermeability and reduced the expression of endothelial glycocalyx, syndecan-1, syndecan-4, perlecan mRNA and heparan sulfate (HS) in the vessels of multiple organs. *In vitro*, a propofol overdose reduced the expression of syndecan-1, syndecan-4, perlecan, glypican-1 mRNA and HS and induced significant decreases in the nicotinamide adenine dinucleotide (NAD+)/NADH ratio and ATP concentrations in human microvascular endothelial cells (HMEC-1). Oligomycin treatment also induced significant decreases in the NAD+/NADH ratio, in ATP concentrations and in syndecan-4, perlecan and glypican-1 mRNA expression in HMEC-1 cells. These results demonstrate that a propofol overdose induces a partially ATP-dependent reduction of endothelial glycocalyx expression and consequently leads to vascular hyperpermeability due to the loss of endothelial barrier functions.

## 1. Introduction

Propofol (2,6-diisopropylphenol) is a widely-used intravenous anesthetic for short-term sedation and general anesthesia during surgery. The clinically optimal concentration of propofol in serum is 2–5 μg/mL for operation [1]. However, an overdose of propofol may abnormally and pathologically cause propofol infusion syndrome (PRIS) accompanied by severe complications in patients with lipemic plasma, fatty liver enlargement, metabolic acidosis, rhabdomyolysis, myoglobinuria and profound hypotension [2]. Propofol concentration-dependently uncouples oxidative phosphorylation and energy production in mitochondria [3]. In addition, a propofol overdose impairs oxygen utilization and inhibits electron flow in the mitochondrial electron transport chain, which decreases ventricular performance [4,5]. However, the detailed molecular mechanisms of PRIS remain unclear. The pathophysiology is complicated by the interactions between a high-dose propofol infusion and underlying critical illnesses, such as systemic inflammatory response syndrome (SIRS) complicated with multiple organ dysfunction syndrome (MODS) [6]. Cytotoxic propofol overdoses in endothelial cells have been hypothesized based on the presence of endothelial injury in SIRS/MODS after intravenous administration of propofol [7,8]. We previously showed that a propofol overdose induces endothelial necrosis-like cell death and vascular barrier dysfunction [9].

Some of the healthy functions of vascular endothelium are maintained via its luminally-coated glycocalyx, which forms the endothelial surface layer with a functional thickness greater than 1 μm projecting from the surface of endothelium into the vessel lumen [10,11]. Endothelial glycocalyx, which consists of membrane-bound proteoglycans and glycoproteins, regulates a number of physiological functions: vascular permeability, fluidic balance, immune response, coagulation and mechanotransduction [12,13,14,15,16]. Among the proteoglycans present in endothelial glycocalyx are syndecans, perlecan and glypicans. There are four subtypes of syndecans and six subtypes of glypicans. Syndecans are found primarily on the cell surface, where each binds a 3–5 heparan sulfate (HS) chain; and perlecan is present in the extracellular matrix [17]. The structure and composition of the endothelial glycocalyx reflect a balance of the biosynthesis of proteoglycans and their shear-dependent removal. Proteoglycans are shed from the endothelial surface in response to reactive oxygen species (ROS) and inflammatory mediators [18]. Their diminutions are associated with a wide range of pathological consequences, for example capillary leak syndrome, edema, aggravated inflammation, platelet hyperaggregation and loss of vascular responsiveness [14].

The loss of vascular tone control might eventually lead to profound hypotension and metabolic acidosis, which are also major manifestations of PRIS. Moreover, considering the vascular dysfunction induced by a propofol overdose [9], the impairment of glycocalyx by a propofol overdose via reducing endothelial energy production has been hypothesized. Therefore, we developed *in vivo* and *in vitro* approaches to investigate the influences of propofol on endothelial glycocalyx. Changes in endothelial HS, proteoglycan core protein (syndecan-1, syndecan-4, perlecan and glypican-1) expression, ATP production and its connections with glycocalyx injury were examined.

## 2. Results and Discussion

### 2.1. Analysis of Vascular Permeability in Propofol-Overdosed ICR Mice

**Figure 1:**
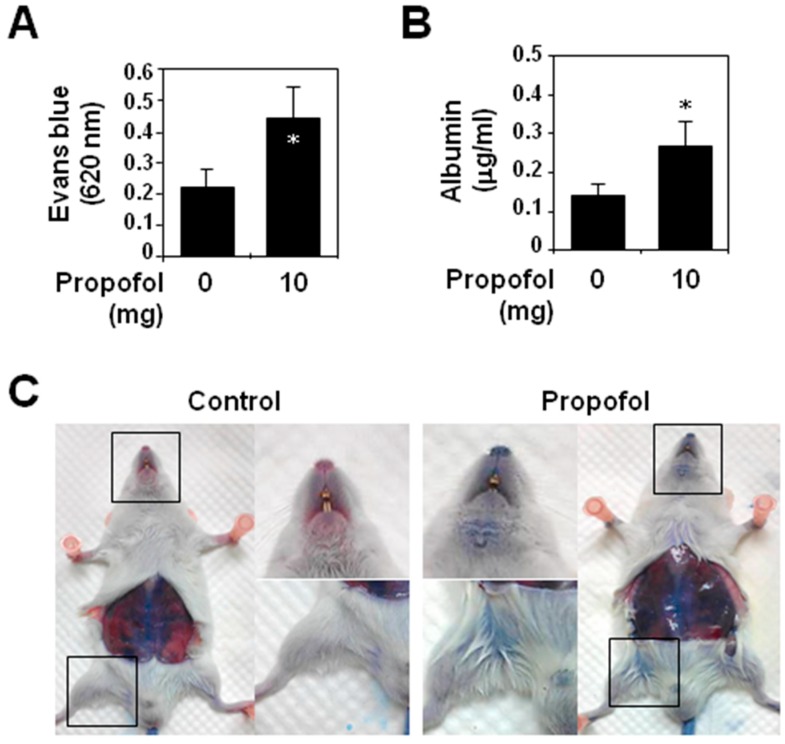
Propofol overdose effect on systemic vascular permeability in ICR mice. Each mouse (*n* = 6 for each group) was intraperitoneally injected with 10 mg of PBS-diluted propofol in the propofol group or with PBS only in the control group within 5 h. (**A**) The optical density (OD) values of peritoneal Evans blue and (**B**) the concentrations of serum albumin in the collected lavaged ascites were analyzed. The values are shown (means ± standard deviation of six mice). * *p* < 0.05, compared with the control group; (**C**) Photographs of mouse snouts and inguinal areas show the degree of Evans blue staining in both groups.

To examine the effects of a propofol overdose in vascular injury, we used the animal model described in Paragraph 2 of the Animal Experiments subsection of the Experimental Section. The vigor of the mice was only mildly depressed throughout the course of propofol injections. Intravenously-injected Evans blue dye, which binds to serum proteins, was used to determine alterations in vascular permeability. Five hours after the initiation of propofol injection, the OD values of Evans blue (Figure 1A) and albumin levels (Figure 1B) in the lavaged ascites of the propofol mice were significantly (*p <* 0.05) higher than those in control mice. In addition, because of the vascular leakage of Evans blue stain, the propofol group mice, but not control group mice, had a significant blue gross appearance, especially in the snout, inguinal area and retroperitoneal wall (Figure 1C). Moreover, mice in the propofol group showed vascular endothelial cells undergoing necrosis-like cell death in transmission electron micrographs (Supplementary Data, Figure S1).

It has been reported that propofol used at therapeutic concentrations has anti-inflammatory and protective effects on vascular endothelium and reduces cerebral edema during ischemic or traumatic insults [19,20]. In this study, we investigated the effects of propofol at doses above the clinically-useful range on vascular endothelium. We found that a propofol overdose significantly increased peritoneal vascular permeability in ICR mice due to propofol-induced necrosis-like cell death of vascular endothelial cells, which supports our previous findings [9]. The effects of an intraperitoneally-administered propofol overdose were not confined to the vasculature within the peritoneal cavity, but were more generalized and caused systemic vascular hyperpermeability in the mice. Although the permeability increases can be simply explained as direct vascular defects due to endothelial cell death, they also occur in blood vessels with damaged endothelial glycocalyx. It is well known that impaired endothelial glycocalyx destroys the vascular barrier function, which leads to protein extravasation and tissue edema and is central in the pathophysiology of ischemia/reperfusion (I/R) injury [21] and many diseases, for example sepsis [22], atherosclerosis [23] and diabetes [24]. The specific role in PRIS is, at this point, not fully known.

### 2.2. Endothelial Glycocalyx Expression in Propofol-Overdosed ICR Mice

**Figure 2:**
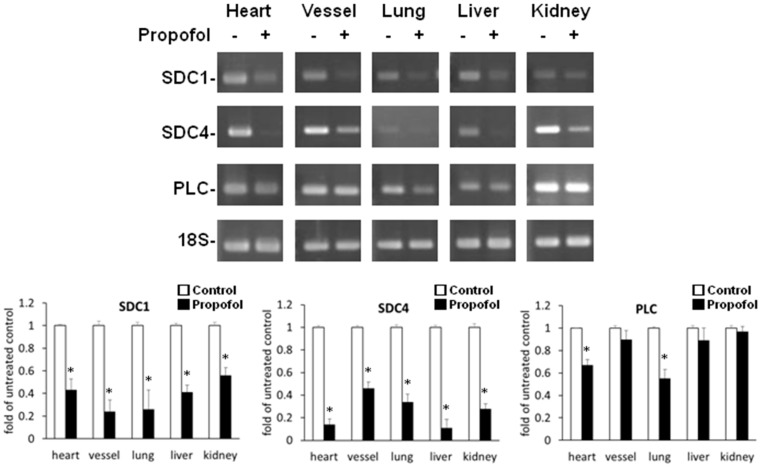
Propofol overdose effect on the relative expression of SDC-1, SDC-4 and PLC mRNA in the various organs of ICR mice. Propofol group mice were intraperitoneally-injected with 10 mg of PBS-diluted propofol within 5 h; the control group mice were injected with PBS only. The relative expression levels of SDC-1, SDC-4 and PLC mRNA of the various organs were detected using RT-PCR. The ratios to the untreated control are shown (means ± standard deviation of six mice). * *p* < 0.05 compared with the control group.

**Figure 3:**
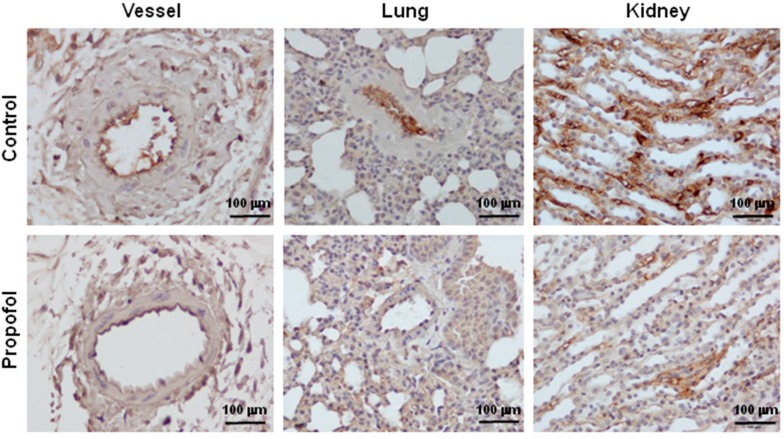
Propofol overdose effect on heparan sulfate (HS) expression of the peritoneal vessels, lungs and kidneys of ICR mice. Propofol group mice were intraperitoneally-injected with 10 mg of PBS-diluted propofol within 5 h; the control group mice were injected with PBS only. The expression of HS was detected using immunohistochemical (IHC) staining of peritoneal vessels, lungs and kidneys, which were then examined using a light microscope.

The relative expression levels of syndecan-1 and syndecan-4 mRNA in heart, blood vessel, lung, liver and kidney tissue and of perlecan mRNA in heart and lung tissue were significantly (*p <* 0.05) lower in the propofol group (Figure 2). Light microscopy after immunohistochemical (IHC) staining of the peritoneal vessel, lung and kidney tissue samples showed that HS was a component of the endothelial glycocalyx. In propofol mice, the endothelial cell lining was markedly less stained (Figure 3).

### 2.3. Endothelial Glycocalyx in Propofol-Overdosed HMEC-1 Cells

**Figure 4:**
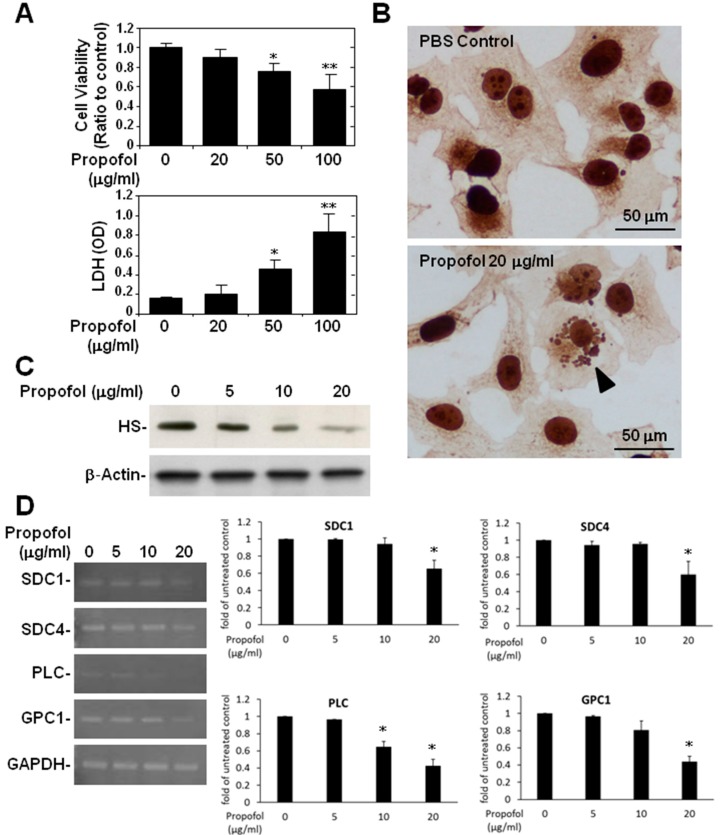
Propofol overdose effect on the relative expression of SDC-1, SDC4, PLC and GPC-1 mRNA and on HS expression in HMEC-1 cells treated with or without propofol for 24 h. (**A**) Cell viability was measured using an MTT assay and cytotoxicity using an LDH assay; (**B**) Endothelial HS expression was detected using immunocytochemical (ICC) staining followed by light microscopy (B, bottom: arrowhead). A micrograph of an apoptotic cell in propofol-treated cells is shown; (**C**) Endothelial HS expression was detected using Western blotting; (**D**) The relative expression levels of SDC-1, SDC4, PLC and GPC-1 mRNA in HMEC-1 were detected using RT-PCR. For the MTT assay and RT-PCR, the ratios to the untreated control are shown (means ± standard deviation of triplicate cultures). * *p* < 0.05, ** *p* < 0.01 compared with the control group. LDH assay data are shown as OD values (means ± SD of triplicate cultures). * *p* < 0.05, ** *p* < 0.01 compared with the untreated group.

An MTT assay showed lower cell viability (Figure 4A, top) and an LDH assay showed greater cytotoxicity (Figure 4A, bottom) in HMEC-1 cells treated with 50 and with 100 μg/mL of propofol. HMEC-1 cells overdosed with propofol showed cell apoptosis (Figure 4B, bottom). To specifically clarify the effect of a propofol overdose on endothelial glycocalyx alone, we lowered the maximum dose of propofol to 20 μg/mL for HMEC-1 cells. Both the Western blot (Figure 4C) and the light microscope images of immunocytochemical (ICC) staining (Figure 4B) showed that endothelial HS was significantly lower in HMEC-1 cells treated with 20 μg/mL of propofol. The full film of another Western blot was shown (Supplementary Data, Figure S2). In addition, RT-PCR showed that the relative expression of syndecan-1, syndecan-4 and glypican-1 mRNA was significantly (*p <* 0.05) lower than in the control cells. The relative expression of perlecan mRNA was even markedly (*p <* 0.05) reduced by propofol at doses above 10 μg/mL (Figure 4D).

In the present study, we also found that a propofol overdose induced a significant reduction of endothelial glycocalyx, both *in vivo*, which subsequently caused systemic hyperpermeability in mice, and *in vitro*. Leakage caused by defects in endothelial barrier functions is one major clinical problem contracted by critically-ill patients; and leads to the severe disturbance of microcirculation, vascular homeostasis and, consequently, the failure of systemic circulation [25,26]. Thus, propofol overdose-induced glycocalyx reduction in vascular endothelium might also contribute to the pathogenesis of PRIS.

Volatile anesthetics, like isoflurane, protect human endothelial cells against cytokine-induced and I/R injury *in vitro* [27]. In addition, preconditioning with sevoflurane effectively decreases coronary leakage [28], hyperemic reaction [29] and leukocyte/platelet adhesion [30] of guinea pig hearts after I/R injury by preserving the endothelial glycocalyx. In contrast, no studies have directly considered the effects of propofol on the glycocalyx. Few studies have investigated the protective effects of volatile anesthetics in general and of propofol in particular on endothelium [31,32]. In fact, one experiment reported by Annecke *et al.* [32] shows an occurrence of glycocalyx shedding with propofol in combination with major surgery, which indicated that propofol somehow aggravates the destruction of glycocalyx. Similar to this finding, the present study is the first to report the post-propofol-overdose reduction of syndecan-1, syndecan-4, perlecan and HS, all constituents of glycocalyx.

That a propofol overdose reduced glypican-1 only *in vitro*, but not *in vivo* is probably because it has tissue characteristics different from those of syndecan, perlecan and HS. The relative expression levels of glypican-1 mRNA in the various organs of the control and propofol groups were below the limit of detection of RT-PCR (data not shown). In addition, the relative expression levels of perlecan mRNA in blood vessel, liver and kidney tissue were not changed by a propofol overdose (Figure 2). Tissue-specific effects of an overdose of propofol on the biosynthesis of particular endothelial proteoglycans might be responsible for these findings.

The actual composition of glycocalyx seems to be a result of the balance between the biosynthesis of proteoglycans and their shedding. Several molecular mechanisms are thought to be responsible for the shedding of the glycocalyx structure, including increases of membrane-bound proteases [33] and metalloproteases (also called metallopeptidases and metalloproteinases) [34], the activation of endothelial heparanase [35] and the generation of ROS by activated inflammatory cells and resident macrophages [36]. Because relatively little is known about the effects of a propofol overdose on glycocalyx, the mechanisms of its diminutions have never been addressed. Cathepsins are ubiquitously expressed proteases, found in all cell types and especially in endothelium [37]. They are stored in lysosomes, but can be easily released by stressed cells and, as such, are associated with degradation of the extracellular matrix. We previously reported [9] that a propofol overdose caused lysosomal membrane permeabilization and subsequent cathepsin D activation, which might be responsible for the destruction of glycocalyx.

### 2.4. ATP Production and Glycocalyx Expression in Propofol-Overdosed HMEC-1 Cells

**Figure 5:**
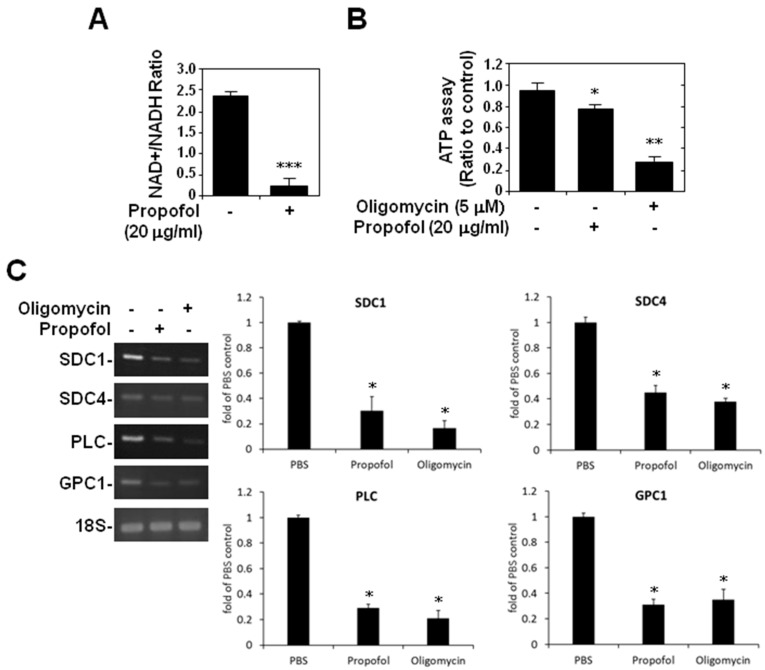
Propofol overdose effect on ATP production and endothelial glycocalyx in HMEC-1 cells. HMEC-1 cells were treated with nothing, propofol or oligomycin for 16 h. NAD+/NADH assay was used to detect the (**A**) NAD+/NADH ratio and an ATP assay to detect (**B**) ATP production in HMEC-1 cells; (**C**) HMEC-1 cells were treated with PBS, propofol or oligomycin for 16 h. The relative expression levels of SDC-1, SDC4, PLC and GPC-1 mRNA in HMEC-1 cells were detected using RT-PCR. The ratios and ratios to the untreated control are shown as means ± SD of triplicate cultures. * *p* < 0.05, ** *p* < 0.01, *** *p* < 0.001 compared with the untreated group.

Because propofol potentially interferes with cellular energy utilization [3] and because of our previous findings of propofol-induced mitochondrial apoptosis [38], we tested the effect of cellular ATP production on endothelial glycocalyx by treating HMEC-1 cells with oligomycin, an ATP synthase inhibitor. The NAD+/NADH assay showed that the NAD+/NADH ratio was significantly (*p <* 0.001) lower in propofol-overdosed cells (Figure 5A), and the ATP assay showed that the ATP concentration was significantly (*p <* 0.05 and *p <* 0.01) lower in both the propofol-overdosed and oligomycin-treated cells (Figure 5B). Moreover, RT-PCR showed that the relative expression of syndecan-4, perlecan and glypican-1 mRNA was significantly (*p <* 0.05) lower in cells overdosed with propofol or treated with oligomycin for 16 h (Figure 5C).

This time, rather than investigating how propofol induces the shedding of glycocalyx, we examined how a propofol overdose reduces its biosynthesis. Consistent with previous studies [5,39], we found that a propofol overdose significantly reduced endothelial ATP concentrations. Moreover, we found that a propofol overdose-induced ATP reduction was associated with a decrease in the production of endothelial proteoglycans. This finding has not been previously reported and is proposed for the first time in the present study. Although ATP depletion might eventually destroy all of the glycocalyx because of its negative effect on cell viability, the concentration of propofol we used here did not cause significant cytotoxicity in HMEC-1 cells. Compared with treatment with oligomycin, an ATP synthase inhibitor, treatment with 20 μg/mL of propofol suppressed only a small portion of the endothelial ATP pool. The noncytotoxic reduction of ATP concentration is, however, sufficient to inhibit endothelial glycocalyx expression. Nevertheless, direct evidence supporting the connection between cellular energy utilization and the homeostasis of endothelial glycocalyx is lacking; thus, additional investigations are required. Because of the similarity of the energy inhibition that occurs in PRIS, we hypothesize that propofol overdose-induced glycocalyx injury is also involved in the pathogenesis of PRIS.

## 3. Experimental Section

### 3.1. Reagents and Antibodies

Propofol and oligomycin were purchased from Sigma-Aldrich (St. Louis, MO, USA) and dissolved in dimethyl sulfoxide. Mouse anti-HS was purchased from Millipore (Billerica, MA, USA), and β-actin-conjugated anti-rabbit immunoglobulin (Ig) G was purchased from Cell Signaling Technology (Beverly, MA, USA). All drug treatments in cells were assessed with cytotoxicity assays before the experiments. Noncytotoxic doses were used in this study.

### 3.2. Cell Cultures

A human microvascular endothelial cell line (HMEC)-1 was provided by Chiou-Feng Lin’s laboratory at the Graduate Institute of Medical Sciences, College of Medicine, Taipei Medical University, Taipei, Taiwan. HMEC-1 cells were cultured in Medium 200 with LSGS (low serum growth supplement), 10% fetal bovine serum (FBS) (GIBCO, Life Technologies, Rockville, MD, USA), 100 µg/mL of streptomycin and 100 units/mL of penicillin (HyClone, Thermo Scientific, Rockford, IL, USA). Cells were maintained in a humidified 5% CO_2_ atmosphere at 37 °C.

### 3.3. Animal Experiments

The 6–8 week-old male progeny of ICR mice were purchased from BioLasco Taiwan (Taipei). The experimental protocol adhered to the rules of the Animal Protection Act of Taiwan and was approved by the Institutional Animal Care and Use Committee of Chi-Mei Medical Center (IACUC Approval No. 102122307).

ICR mice (*n* = 6 per group) were given 5 intraperitoneal (i.p.) injections of propofol (2 mg) diluted in phosphate-buffered saline (PBS) (total volume: 0.2 mL) at 1-h intervals (propofol group). Control mice were given injections (i.p.) of PBS only (0.2 mL) at the same time (control group). Any changes in the activity of the mice in both groups were recorded throughout the course of injections. Thirty minutes after the last dose of propofol or PBS, each mouse was injected intravenously (i.v.) with 200 μL of 1% Evans blue dye solution. A peritoneal lavage with 3 mL of PBS was done 30 min later, and the optical density (OD) values of the Evans blue dye and the concentrations of serum albumin in the collected lavaged ascites were analyzed using a microplate reader (Spectra MAX 340PC; Molecular Devices, Sunnyvale, CA, USA) with excitation at 620 nm and emission at 680 nm, using an automatic biochemical analyzer (7080; Hitachi Koki Co., Tokyo, Japan), respectively. Thirty minutes after the peritoneal lavage, the mice were killed, and their hearts, lungs, livers, kidneys and peritoneal vessels were collected for histological and biochemical analysis.

### 3.4. Reverse Transcriptase-Polymerase Chain Reaction

Total RNA was extracted from the mouse hearts, lungs, livers, kidneys and peritoneal vessels using a reagent (TRI; Sigma-Aldrich), and then, the total RNA underwent reverse transcription. Syndecan-1, syndecan-4, perlecan and glypican-1 were amplified using PCR with gene-specific primers (Table 1). PCR products were visualized on 1.5% agarose gels containing ethidium bromide. 18S and glyceraldehyde 3-phosphate dehydrogenase (GAPDH) amplification was used as an internal control. The RT-PCR data were analyzed using ImageJ software (Version 1.41o) (National Institutes of Health, Bethesda, MD, USA) (http://rsbweb.nih.gov/ij/), and results are expressed as fold differences.

### 3.5. Viability Assay

We used an MTT (3-[4,5-dimethylthiazol-2-yl]-2,5-diphenyltetrazolium bromide; Sigma-Aldrich) assay to evaluate the effect of propofol on cell viability. The cells were cultured in 96-well tissue culture plates in maintained medium with propofol-treated cells. The cells were then incubated with MTT solution for 3 h. The supernatant was aspirated, and DMSO (dimethyl sulfoxide) (Sigma-Aldrich) was added to dissolve the blue crystals. The microplate reader was used to measure the absorbance at 450 nm, and the data were analyzed using Softmax Pro software (Molecular Devices). Cell viability was evaluated by the percentage of the number of live cells in the treatment group relative to the number in the control group [40].

**Table 1:** Primers for real-time polymerase chain reactions.

Gene	Forward	Reverse
18S	AGCTCCCGAAAGCGACGTT	ATCTTGCAAAGCACCTGCAC
GAPDH	GGCGATGCTGGCGCTGAGTA	ACAGTTTCCCGGAGGGGCCA
SDC-1 (human)	GAGTTCTACGCCTGATGGGG	CGACAGGTGTGGTTGTGGTA
(mouse)	GGACCTCCTAGAAGGCCGATA	AGTTTCTTGGGTTCGGTGGG
SDC-4 (human)	CCCGGAGAGTCGATTCGAGA	GAGCTGCCAAGACCTCAGTT
(mouse)	TTGAGCTCGTCCCACAACGA	TGGTTCAGAGCACCAGGTTG
PLC (human)	CACACACCGACCACATACCA	CTCTGCCTAGCGATTCTGGG
(mouse)	AGTTCTGGGGTACATCGGGT	TCATGTCCGGCTTGGTGATG
GPC-1 (human)	CGGCTTTTGTTGTCTCCGC	GCATATAGGTCCCGGAAGGC
(mouse)	AGCTCCCGAAAGCGACGTT	ATCTTGCAAAGCACCTGCAC

### 3.6. Cytotoxicity Assay

To evaluate cell damage, lactate dehydrogenase (LDH) activity was assayed using a colorimetric assay (Cytotoxicity Detection kit; Roche Diagnostics, Lewes, UK). Aliquots of the culture media were transferred to 96-well microplates. The microplate reader was used to measure the absorbance at 620 nm with a reference wavelength of 450 nm, and the data were analyzed using Softmax Pro.

### 3.7. Immunohistochemistry and Immunocytochemistry

Paraffin-embedded mouse-tissue samples and HMEC-1 cell cultures were stained for HS using anti-HS monoclonal antibody (MAB2040; Millipore) and analyzed by light microscopy. Incubating paraffin-embedded mouse-tissue sections with mouse IgG1 isotype (clone 11711; R&D Systems, Minneapolis, MN, USA) instead of anti-HS monoclonal antibody was the negative control.

### 3.8. Western Blotting

Harvested cells were lysed with a buffer containing 1% Triton X-100, 50 mM Tris (pH 7.5), 10 mM EDTA, 0.02% NaN_3_ and a protease inhibitor cocktail (Roche Boehringer Mannheim Diagnostics, Mannheim, Germany). After one freeze-thaw cycle, the cell lysates were centrifuged at 10,000× *g* at 4 °C for 20 min. The lysates were then boiled in sample buffer for 5 min. The proteins were subjected to sodium dodecyl sulfate polyacrylamide gel electrophoresis (SDS-PAGE) and transferred to a polyvinylidene fluoride (PVDF) membrane (Millipore, Billerica, MA, USA) using a semi-dry electroblotting system. After they had been blocked with 5% skim milk in PBS, the membranes were incubated with a 1/1000 dilution of anti-HS antibodies at 4 °C overnight. The next day, the membranes were washed with 0.05% PBS-Tween 20 and incubated with a 1/5000 dilution of horseradish peroxidase-conjugated secondary antibodies at room temperature for 1 h. After they had been washed, the membranes were soaked in an enhanced chemiluminescent (ECL) substrate solution (PerkinElmer Life Sciences, Boston, MA, USA) for 1 min and were then exposed to film (BioMax; Eastman Kodak, Rochester, NY, USA).

### 3.9. NAD+/NADH Assay

A nicotinamide nucleotide assay was done using a kit (NAD+/NADH Quantification kit; BioVision, San Francisco, CA, USA). Equal amounts of cell lysate were collected and homogenized with NAD/NADH extraction buffer. The lysates were centrifuged at 14,000 rpm for 5 min at 4 °C. The supernatant was collected for a colorimetric (λ_max_ = 450 nm) analysis of NAD+/NADH using NAD cycling mix.

### 3.10. ATP Assay

Detection and analysis of the total ATP content of propofol-treated HMEC-1 cells were done using an ATP determination kit (BioVision). Briefly, an equal number of cell lysates were collected and lysed in ATP assay buffer. The lysates were centrifuged at 15,000× *g* for 2 min at 4 °C. The supernatant was collected for a colorimetric (λ_max_ = 570 nm) analysis of ATP using phosphorylated glycerol kinase. Relative ATP content was calculated based on the peak area *versus* the ATP standard curve.

### 3.11. Transmission Electron Microscopy

Vascular endothelial tissue samples were fixed in 2% glutaraldehyde and 2% paraformaldehyde in 0.1 mol·L^−1^ sodium cacodylate buffer, cut into small pieces and maintained at 4 °C until further processing. The fixed tissue samples were washed three times in 0.1 mol·L^−1^ of sodium cacodylate buffer, osmium tetroxide, double deionized water and EtOH, in which they were allowed to sit overnight at 4 °C. They were infiltrated and washed three times in Embed-812 resin (with and without Tris-(dimethylaminomethyl) (DMP-30)) and 100% EtOH and then allowed to sit overnight. The tissue samples were then infiltrated with fresh resin, which was set in BEEM embedding capsules and allowed to polymerize for 48 h at 70 °C. Sections of vascular endothelial tissue in polymerized resin were visualized and analyzed using a transmission electron microscope.

### 3.12. Statistical Analysis

Values are expressed as the means ± standard deviation (SD). Groups were compared using the Mann–Whitney *U*-tests or Kruskal–Wallis tests. Statistical significance was set at *p* < 0.05.

## 4. Conclusions

In conclusion, using a mouse model and an HMEC-1 cell line, we showed the negative effects of a propofol overdose on vascular barrier functions and on endothelial glycocalyx, both *in vivo* and *in vitro*. We partially defined the molecular mechanisms of propofol-induced glycocalyx reduction by using a noncytotoxic dose of propofol (20 μg/mL). All of the results suggest that a propofol overdose reduces endothelial glycocalyx and leads to vascular hyperpermeability because of the loss of endothelial barrier functions. The interference with ATP utilization in endothelial cells caused by a propofol overdose may partly explain the glycocalyx reduction. These findings show the potential deleterious side effects of the long-term clinical use of large doses of propofol on vascular endothelium.

## Supplementary Materials

Supplementary materials can be found at http://www.mdpi.com/1422-0067/16/06/12092/s1.
