# Supplementary material for: Anesthetic Propofol Overdose Causes Vascular Hyperpermeability by Reducing Endothelial Glycocalyx and ATP Production
Source: Int J Mol Sci. 2015 May 27;16(6):12092–107. doi: 10.3390/ijms160612092 (PMC4490431; doi:10.3390/ijms160612092)
Supplement: Supplementary file 1 [file ijms-16-12092-s001.pdf]

Supplementary Information

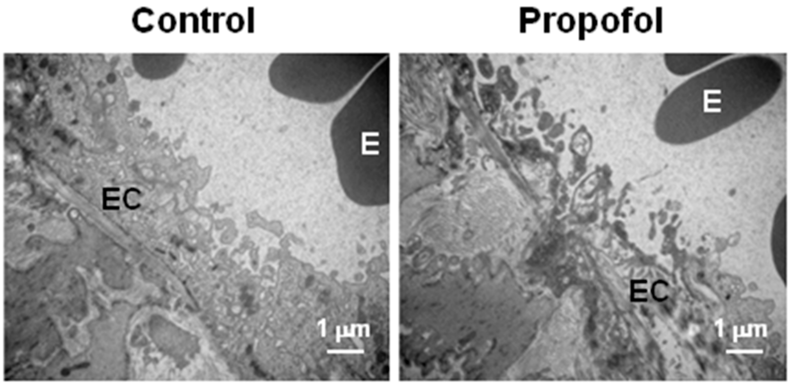

Figure S1. Propofol overdose caused vascular endothelial cells to undergo necrotic cell death in ICR mice. Propofol group mice were intraperitoneally injected with 10 mg of PBS-diluted propofol within 5 h; control group mice were injected with PBS only. Loss of plasma membrane integrity and cytoplasm was detected using transmission electron microscopic images of the peritoneal vascular endothelium. EC, endothelial cell; E, erythrocyte.

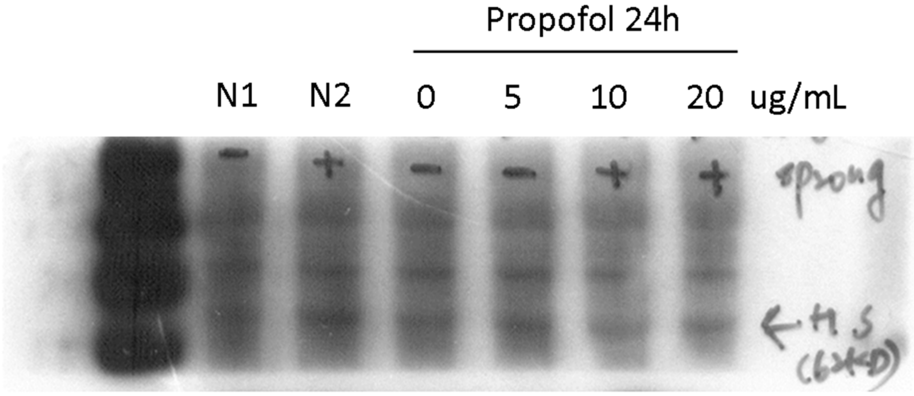

Figure S2. HMEC-1 cells treated with or without propofol for 24 h. Endothelial HS expression was detected using Western blotting, as described in the Experimental Section. N1 and N2: negative controls.
